# Supplementary material for: Therapeutic Targeting of Ovarian Cancer Stem Cells Using Estrogen Receptor Beta Agonist
Source: Int J Mol Sci. 2022 Jun 28;23(13):7159. doi: 10.3390/ijms23137159 (PMC9266546; doi:10.3390/ijms23137159)
Supplement: Supplementary file 1 [file ijms-23-07159-s001.zip › ijms-1766947-supplementary.pdf]

Supplementary Fig. S1

A

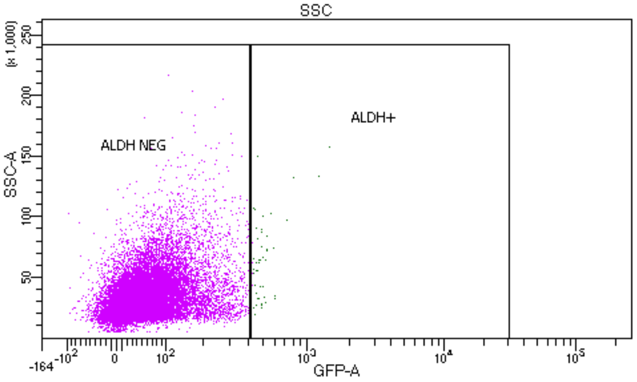

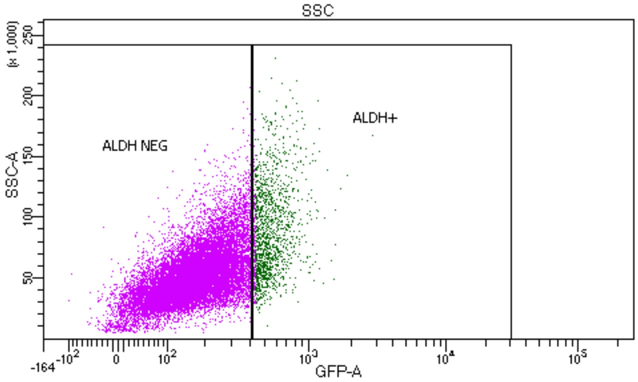

Tube: SKOV3 DEAB - CONTROL

Population	#Events	%Parent	%Total
All Events	20,294	####	100.0
SCATTER	20,137	99.2	99.2
FSC	20,112	99.9	99.1
SSC	20,000	99.4	98.6
ALDH+	51	0.3	0.3
ALDH NEG	19,955	99.8	98.3

Tube: SKOV3 ALDH SAMPLE

Population	#Events	%Parent	%Total
All Events	20,490	####	100.0
SCATTER	20,361	99.4	99.4
FSC	20,316	99.8	99.2
SSC	20,000	98.4	97.6
ALDH+	1,530	7.6	7.5
ALDH NEG	18,641	93.2	91.0

B

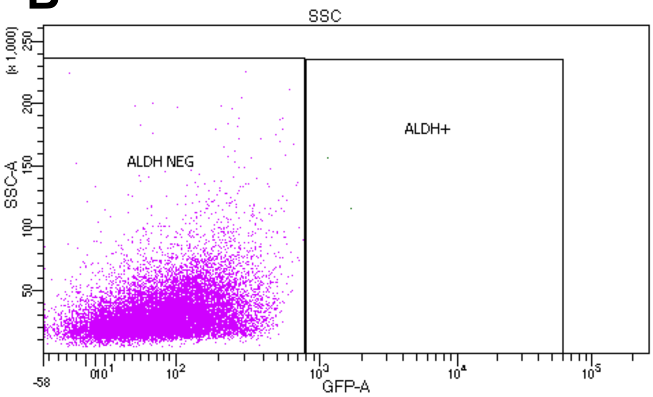

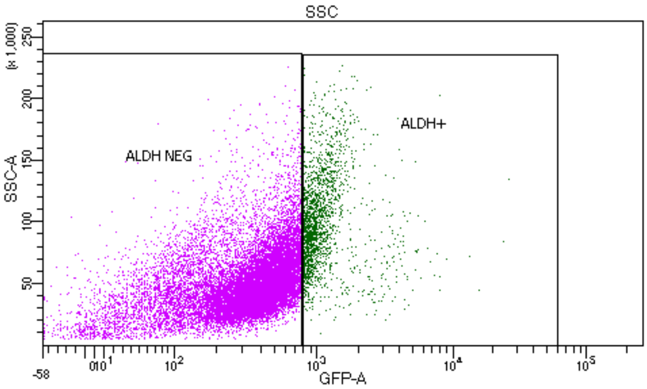

Tube: DEAB - CONTROL

Population	#Events	%Parent	%Total
All Events	20,400	####	100.0
SCATTER	20,249	99.3	99.3
FSC	20,237	99.9	99.2
SSC	20,182	99.7	98.9
ALDH+	2	0.0	0.0
ALDH NEG	20,180	100.0	98.9

Tube: A2780 ALDH SAMPLE

Population	#Events	%Parent	%Total
All Events	20,745	####	100.0
SCATTER	20,311	97.9	97.9
FSC	20,276	99.8	97.7
SSC	20,000	98.6	96.4
ALDH+	1,870	9.4	9.0
ALDH NEG	18,130	90.6	87.4

Supplementary Figure S1: Gating strategy for defining ALDH-ve and ALDH+ve cells. Representative flow cytometry images were shown for SKOV3 (**A**) and A2780 (**B**). DEAB that inhibits the ALDH activity was used as negative control in order to set the gate of the ALDH+ve population.

Supplementary Table S1

Gene	SKOV3_Veh_	SKOV3_Veh_	SKOV3_LY_1	SKOV3_LY_2	log2FoldChange	p-value
KCND1	432	462	187	189	-1.249854989	2.65E-08
AGER	528	511	247	169	-1.320939417	3.28E-08
CDKN1A	8261	8550	13057	14739	0.725407582	5.37E-08
ZNF117	1748	1786	1172	826	-0.821866264	8.67E-06
DNHD1	1176	991	547	673	-0.829205778	8.71E-06
ANXA1	311	337	584	689	0.974925815	1.67E-06
RMRP	12	2	56	86	3.257290643	3.20E-06
KHDC1L	528	487	759	1282	1.008067029	1.33E-05
PLTP	3518	3793	5087	6142	0.619249265	2.76E-05
SERTAD1	658	536	832	1595	1.023276539	4.90E-05
SESN2	2195	2187	2985	3967	0.665560008	5.23E-05
NDUFAF3	846	882	1218	1641	0.726676147	7.15E-05
MIR3648	124	105	168	489	1.518982452	8.95E-05
PRRT2	6039	7071	4522	3208	-0.761946127	1.25E-05
C4orf21	1538	1429	953	898	-0.680781962	1.61E-05
STC2	1581	1767	1054	1062	-0.66255823	2.09E-05
RPS27L	3022	2905	3938	5033	0.597702177	0.00012932
DOK3	264	198	102	87	-1.2921827	2.30E-05
NCRNA00263	796	882	527	464	-0.758978282	3.75E-05
LGALS1	4160	4230	5257	9173	0.782345241	0.00014112
ATP6V0B	746	803	1003	1945	0.928328981	0.00014135
TP53I3	1930	2050	2749	3070	0.548028931	0.00016498
C4orf47	105	105	36	23	-1.827703048	4.23E-05
C19orf20	289	271	419	633	0.908299128	0.00016756
C9orf16	1443	1486	1982	2469	0.603401178	0.00018499
FDXR	1555	1207	2049	2210	0.624217959	0.00019395
VGF	11942	11527	15340	18142	0.512611489	0.00020558
AHNAK	3265	2955	4368	4497	0.511100062	0.00021272
NINJ1	1259	1089	1770	1734	0.577420593	0.00023327
TNFSF9	455	467	672	846	0.719030458	0.00023765
ATP5D	1784	1944	2535	2947	0.556149478	0.00024224
PCSK1N	401	498	656	879	0.77287477	0.00027477
ITPA	881	667	1098	1467	0.727800071	0.00028377
PLAU	1226	1239	1741	1851	0.543387121	0.00029647
PROCR	880	924	1332	1347	0.570676668	0.00029882
ADM	426	433	215	248	-0.892141849	6.37E-05
ANKRD36	794	805	531	406	-0.770460301	9.01E-05
INTS5	529	520	728	1012	0.730818346	0.00031686
C12orf45	221	303	459	472	0.82947715	0.00033075
NDUFA2	628	542	741	1485	0.928228823	0.00033302
MRPL14	1030	898	1314	1665	0.627226106	0.00039245

SLC34A2	897	895	1305	1334	0.558166466	0.00039398
RPL18A	88	74	116	338	1.487484382	0.00039962
HMOX1	1080	1058	1404	1914	0.633478077	0.0004407
CILP2	513	522	709	989	0.714143732	0.00046748
TXNRD2	638	652	856	1259	0.712536761	0.00049148
BBC3	1384	1354	1830	2169	0.546166045	0.00049594
PDK1	1738	1698	1082	1190	-0.59740849	9.92E-05
SLC26A10	202	188	81	89	-1.198926164	0.00010311
RPLP2	4451	5137	5805	9732	0.696447687	0.00054575
MIR663	30	33	57	164	1.804937529	0.0005495
C17orf70	1008	979	1275	1897	0.674740585	0.00055055
RHBDD2	2097	1939	2644	3189	0.531247405	0.00057489
LAGE3	229	298	344	742	1.044003326	0.00060469
CFH	68210	66266	50750	40261	-0.563231186	0.00010396
TBL3	864	1032	1235	1796	0.676477737	0.00066303
PAN3-AS1	324	272	169	105	-1.119522313	0.00012868
RABAC1	793	736	974	1547	0.720855539	0.00069975
FAM176B	965	846	1094	2085	0.81135786	0.00072812
TRIAP1	1537	1444	1940	2423	0.549340929	0.00073447
MZT2B	908	842	1050	2019	0.809712296	0.00077487
TRIM48	65	60	157	130	1.192534982	0.00083178
ISG15	444	460	597	924	0.749749226	0.00084561
HSPA1B	926	848	1175	1499	0.592170067	0.00085166
RBX1	862	1152	1288	2052	0.729596084	0.00088648
NDUFS3	2084	1995	2539	3565	0.581443775	0.00088671
GNB2	3322	3126	3990	5504	0.55786398	0.00090449
COX8A	2026	2122	2432	4409	0.721593343	0.00092379
MYBL1	685	741	470	367	-0.767795555	0.00013051
SURF1	336	286	432	648	0.796831039	0.00092458
YRDC	503	427	616	949	0.750046248	0.00093789
METRN	300	376	442	799	0.876583548	0.00098235
ATP5O	2851	2825	3376	5492	0.643550542	0.00102962
CCDC18	1209	1126	815	519	-0.807127382	0.00013404
HK2	4892	4711	3487	3217	-0.518574987	0.00013719
C3orf70	336	401	194	199	-0.905849934	0.00014307
GSDMB	577	682	394	342	-0.772601737	0.00014497
C16orf53	5600	5752	4191	3755	-0.514558575	0.00016462
AGTRAP	287	280	386	612	0.815792144	0.00107053
C16orf5	1410	1176	1709	2107	0.56069709	0.0010745
C12orf57	1166	1092	1418	2030	0.610631273	0.00113773
NCRNA00201	4812	5124	3655	3258	-0.523241617	0.00017582
ZNF160	3467	3508	2608	1950	-0.613802937	0.00019426
ZNF138	1820	1652	1260	831	-0.73129381	0.00020661

SNAP47	1427	1487	1843	2440	0.55488423	0.00114959
ATP6AP1	2958	3129	3795	4894	0.513342771	0.0011585
MRPL41	1101	1018	1341	1888	0.606874454	0.00116173
IFI27L2	288	253	399	505	0.741177176	0.00117396
DPCD	355	449	568	743	0.704749889	0.00118279
SMC4	16377	15988	12416	8731	-0.613918803	0.00022945
LOC10049917	2874	3015	2200	1778	-0.565642719	0.00028762
FAM158A	356	349	507	615	0.668330524	0.00121496
NCRNA00085	555	712	408	334	-0.77194008	0.00032163
ZNF121	2565	2391	1863	1380	-0.61193154	0.00036381
CASC5	3342	3554	2610	2158	-0.532205278	0.00044068
SH3D21	783	900	570	505	-0.64631958	0.00045527
SGOL2	2305	2241	1646	1552	-0.507749714	0.00045669
TRIP11	3227	2945	2352	1741	-0.59212623	0.00048434
MAMDC4	309	314	148	188	-0.888906733	0.00049513
PAR5	1154	1338	835	836	-0.576430123	0.00052633
MAP1LC3A	116	132	200	283	0.958226244	0.00122844
EMP3	2639	2760	3244	4936	0.599225043	0.00123421
INPP5D	763	796	1064	1206	0.541957588	0.00124741
CENPJ	2718	2918	2158	1512	-0.61876311	0.00053439
CENPE	5968	5850	4610	3293	-0.58023737	0.00054521
DUSP2	110	110	167	290	1.055701796	0.00127536
C19orf56	720	726	835	1731	0.826974013	0.00130121
NR4A2	2354	2559	1862	1486	-0.553304897	0.00061562
CEP55	1446	1556	1124	839	-0.612831396	0.00065803
PNISR	21864	22998	17776	10586	-0.661498985	0.00071345
F11R	880	1029	1295	1490	0.54460478	0.00130541
CENPC1	1212	1185	868	760	-0.558106031	0.00077386
CP	5216	5025	4004	3187	-0.510062539	0.00078972
TMEM147	1098	974	1288	1948	0.642204944	0.00131829
ALPPL2	161	182	301	299	0.804401361	0.00132099
RPL29	4742	4801	5747	8148	0.542016799	0.00135939
RFPL4B	450	456	667	700	0.593654552	0.0013642
FAM173A	236	170	285	492	0.93453917	0.00137157
B3GAT3	525	515	677	980	0.671157946	0.00138643
NUDT19	293	316	428	577	0.721478771	0.00140557
TMEM59L	75	74	138	178	1.089940439	0.00145552
CCNA1	216	264	342	498	0.807200994	0.00145805
ZNF354B	1694	1606	1261	724	-0.732475833	0.00086545
JHDM1D	1761	1716	1313	1088	-0.534113952	0.00093048
C9orf23	399	331	499	705	0.722069318	0.00147868
LOC10050612	923	986	710	418	-0.758782797	0.00093845
SGK494	795	856	545	557	-0.581833116	0.00100136

MIS18BP1	2342	2331	1801	1471	-0.513841349	0.00107652
INSIG2	1142	1015	707	761	-0.55526109	0.00108295
NCRNA00202	124	134	60	51	-1.208491899	0.00112068
FAS	545	515	724	881	0.597850752	0.00151398
TPRA1	463	482	629	857	0.651986879	0.0015289
SSSCA1	313	330	447	599	0.703301801	0.00153641
JMJD4	786	723	948	1413	0.645114317	0.00153966
STOML1	26	26	67	89	1.568462916	0.00154043
WDR52	536	415	323	224	-0.798390899	0.00122894
COL7A1	735	827	555	465	-0.613888994	0.00123782
ATP13A2	1184	1182	1506	1927	0.53656544	0.00154073
ESPNL	264	287	415	473	0.690019061	0.00157282
CIB1	506	414	596	928	0.726522247	0.00168449
C7orf68	1136	1433	902	828	-0.57008072	0.00124658
SARNP	873	900	1106	1597	0.608468953	0.00168631
TAP1	215	216	305	449	0.805369108	0.00170342
USMG5	1553	1508	1872	2643	0.560214327	0.0018199
CENPM	155	183	214	481	1.039060936	0.00182455
FAM125A	552	512	711	905	0.60327695	0.00185772
CHCHD10	1573	1505	1925	2475	0.515366384	0.00188496
LUC7L3	18386	20275	15548	11418	-0.519675713	0.00127417
UCP2	1986	1813	2351	3110	0.522944967	0.0018955
NRBP2	1244	1152	860	809	-0.52131163	0.00138671
SNORD57	92	82	34	30	-1.447427421	0.00141056
ZNF37BP	6479	6136	5091	3404	-0.570273166	0.00145943
PSENEN	661	702	893	1119	0.562056186	0.00208058
C1orf122	546	465	622	1069	0.741297469	0.00208938
ZCCHC12	397	383	521	714	0.661412764	0.0020978
CIAPIN1	1078	1024	1247	2051	0.649432994	0.00211802
CALML4	815	789	583	479	-0.593271662	0.00151019
GPX1	1614	1693	2018	2786	0.538495733	0.00212368
ERV3-1	626	633	425	406	-0.599954321	0.00153253
BTNL9	503	571	386	253	-0.748622486	0.0016822
UBL5	833	829	945	1853	0.750712137	0.00219564
PRAMEF12	188	219	311	372	0.749401939	0.00223018
SCN4B	213	187	324	340	0.73230302	0.00230647
CA2	187	243	355	361	0.737411571	0.00234642
ERG	642	676	484	339	-0.677070205	0.00169358
RIMKLB	2132	2014	1619	1288	-0.512073691	0.0017263
RPL4	11137	10107	11838	21039	0.629932444	0.00236495
CD81	2180	2107	2572	3628	0.532121129	0.00237786
NSUN6	655	649	464	392	-0.605156769	0.00181629
GNB3	244	223	140	114	-0.877372591	0.0018909

GPN2	361	413	516	709	0.663346702	0.00243594
ARPC4	1377	1419	1683	2451	0.56402796	0.00244544
HLA-DOA	619	532	843	838	0.546358773	0.00244626
MIR1281	92	123	55	19	-1.53331063	0.00190206
TCEB2	2171	2367	2670	4080	0.572480126	0.00245443
ISOC2	838	869	1047	1527	0.592450463	0.00256508
PSMB3	2143	2176	2519	3915	0.574853256	0.00260708
CHID1	1437	1502	1678	2931	0.649390242	0.0026259
TST	243	258	323	556	0.810371211	0.00263518
ZNF92	1697	1662	1301	1064	-0.505854846	0.00196442
TLCD1	333	381	500	606	0.629842364	0.00270172
LOC284801	131	169	195	406	1.002243283	0.00274036
NUDT16L1	480	414	546	935	0.727773723	0.00289027
FBXO43	577	613	428	334	-0.641792595	0.00200942
TYSND1	1031	937	1226	1641	0.542570494	0.00291241
SLC35B1	784	751	920	1463	0.633051431	0.0029513
GLS2	504	491	695	756	0.543869025	0.00297992
COQ10A	391	398	553	628	0.583186928	0.00303621
BCL2L12	449	429	556	827	0.654806123	0.0030918
KIAA1797	1468	1452	1758	2440	0.523375692	0.00321834
C22orf32	357	346	424	777	0.771090448	0.00324696
LRRC24	101	131	159	310	1.017676407	0.0033146
C2orf63	399	411	233	269	-0.690036751	0.002013
C11orf2	1388	1381	1639	2427	0.553997872	0.00336303
HPS6	448	481	608	795	0.59448028	0.00336804
MIR641	1	1	5	38	4.328516962	0.00339491
ARHGAP33	419	513	290	296	-0.66910689	0.00214917
ZNF525	824	715	553	474	-0.582205773	0.00217025
RPS21	4035	3767	4550	6568	0.51071306	0.00343381
ATOX1	394	435	511	833	0.697372725	0.00343456
MNS1	325	328	225	142	-0.832254699	0.0023282
ATP5G2	3527	3301	3984	5754	0.512065855	0.00352242
NUF2	1650	1416	1158	999	-0.507078388	0.00238228
PDCL3	418	372	578	597	0.571776353	0.00352687
MFSD3	703	614	848	1087	0.554268577	0.003533
GOLGA2B	989	1025	762	621	-0.541551088	0.00243083
GKAP1	596	639	442	374	-0.598071823	0.00249139
HSPBP1	588	613	746	1069	0.595660755	0.00357209
MMP11	316	337	426	616	0.674552898	0.00360122
SCAND1	900	781	956	1738	0.680251869	0.00381505
NAMPT	3779	3794	3106	1998	-0.568883598	0.002575
ZC3H6	526	542	378	315	-0.621793811	0.00260062
PLA2G15	263	275	356	519	0.702719236	0.00386321

PNN	20029	21843	17466	10753	-0.569304436	0.00281906
EIF6	1363	1269	1530	2400	0.57808733	0.00388397
MAD2L1BP	975	964	1190	1590	0.519313853	0.00400319
S100A4	910	885	1012	1823	0.658997973	0.00411685
ORAI3	976	968	1194	1580	0.513428388	0.00412859
C20orf4	926	872	1100	1500	0.532324811	0.00419719
NTPCR	709	684	822	1320	0.620758217	0.00425185
ZSCAN4	337	338	500	506	0.576588321	0.00432433
MAD2L2	812	755	953	1359	0.560319585	0.00433455
ZNF107	4120	4378	3492	2388	-0.531284804	0.00285544
PEX16	228	194	286	426	0.754279804	0.00436162
CRYL1	109	112	164	250	0.902143967	0.00447435
ROMO1	1023	904	1163	1655	0.547197912	0.00458166
PSMA2	2914	2757	3107	5461	0.595096611	0.00463671
C7orf59	1297	1262	1516	2156	0.520992632	0.00464325
TMEM59	2317	1648	2482	3297	0.543253452	0.00468171
NDUFC2	350	357	430	718	0.699084399	0.00480657
NENF	916	856	1055	1557	0.559257369	0.00483555
NDUFB11	1867	1955	2233	3184	0.50304903	0.00485517
ZNF85	799	884	651	428	-0.640983618	0.00300822
B9D1	198	215	248	503	0.860706798	0.00490572
ANKRD18A	1803	2077	1561	1050	-0.570708424	0.00319713
MCOLN1	596	544	784	830	0.50117285	0.00500351
ANKRD30BL	0	0	12	9	5.696857068	0.00507736
PNMAL2	100	102	211	159	0.877024487	0.00542002
CMTM3	1934	1990	2188	3593	0.558567878	0.00560544
CLP1	646	730	846	1166	0.546994182	0.0056092
TBRG4	2196	2213	2528	3710	0.50026801	0.00576369
YIF1B	500	458	608	809	0.564738224	0.00577239
PEMT	369	305	488	522	0.581553518	0.00577486
COBLL1	922	944	714	573	-0.535451291	0.00338628
GIPR	708	775	559	445	-0.561644656	0.00394948
TMED9	1971	1909	2229	3278	0.504870759	0.00587607
CALML6	88	108	51	29	-1.284929139	0.00422269
LOC388789	672	711	811	1270	0.589021214	0.00592104
ATP5G1	670	721	797	1342	0.621129194	0.00592279
JOSD2	77	60	101	193	1.094439633	0.00599911
UPF3A	1358	1420	1123	777	-0.547452436	0.00429818
COL27A1	1022	1024	817	553	-0.577451799	0.00441157
C17orf61	702	624	787	1201	0.583272368	0.0061719
RPL34	2144	2062	2258	4107	0.597569542	0.0062612
ZNF606	815	927	619	610	-0.502132417	0.00449024
RPS23	3196	2820	3289	5612	0.565119045	0.00626479

ASPHD1	533	450	565	1013	0.682043482	0.00629222
DDX28	586	601	762	918	0.501238267	0.00633758
MMP14	528	525	365	346	-0.565158647	0.00457159
LOC642361	138	124	72	56	-1.034986543	0.00457276
COMMD4	892	759	910	1696	0.657497851	0.00634459
PABPN1	1745	1677	1414	840	-0.602518351	0.00461626
GAMT	705	635	804	1174	0.56153518	0.0063447
TMEM198	194	208	280	369	0.69135809	0.00635541
RERGL	44	44	12	13	-1.854037426	0.00471515
NME3	444	424	460	1033	0.781984489	0.00641545
GAS2L3	653	518	444	290	-0.672932795	0.00500634
RSRC2	9166	9895	8139	4784	-0.560717205	0.00510743
UBA52	4891	4603	5190	8553	0.533417678	0.00648346
ZNF214	408	343	252	227	-0.650155553	0.00517675
DCTPP1	656	694	779	1269	0.601274481	0.00650366
SLC25A19	252	280	402	404	0.59862151	0.00650449
COBRA1	1583	1465	1764	2562	0.504476215	0.00659615
ZNF595	1728	1504	1338	688	-0.673429839	0.00523008
C12orf62	447	496	598	790	0.557220425	0.00660441
UBE2Q2P1	101	114	47	53	-1.107315953	0.00524278
ZNF493	591	729	498	378	-0.591788208	0.00554152
SNX17	1772	1632	1859	3240	0.582427232	0.00663238
EBF3	519	579	370	381	-0.548853341	0.00579435
ZNF429	388	376	284	180	-0.714551327	0.00590309
PROCA1	317	360	237	187	-0.675229859	0.00608656
RPGR	591	593	448	355	-0.559967114	0.00611747
MSH5-C6orf2	83	103	41	41	-1.168880937	0.00631726
RBM25	19491	20590	17325	10863	-0.507770215	0.00650764
EFNB1	572	732	892	960	0.506603262	0.00667498
TCERG1	19792	20271	17342	10915	-0.503625207	0.00662527
PI4KAP1	112	132	66	55	-1.01473018	0.00666336
PPP2R4	5576	5248	5977	9429	0.509155694	0.00668546
AURKC	71	56	21	26	-1.440760862	0.00667416
MAP2K3	400	520	572	844	0.622824225	0.00669535
ZNF675	1381	1429	1167	775	-0.532132269	0.00710716
PIGO	931	850	1044	1551	0.542153052	0.00674578
MTFMT	350	378	519	540	0.541127421	0.00677809
PPFIA4	553	509	330	392	-0.555053498	0.00716553
RPS15A	2789	2809	3125	4837	0.508002595	0.00687381
KCNAB3	327	288	199	193	-0.651099353	0.00765558
ZNF25	737	723	576	434	-0.530627302	0.0079003
C19orf70	470	465	600	751	0.532104946	0.00706257
CCR10	445	460	528	883	0.640807065	0.00715932

RNF5P1	143	121	171	317	0.88422669	0.00716682
YBX2	1122	1341	1020	633	-0.574788913	0.00824473
MURC	329	292	215	180	-0.652186281	0.00856677
TPRG1L	451	438	583	695	0.523297467	0.00742314
C15orf37	103	97	183	171	0.824484053	0.00748756
SNORD29	144	171	94	78	-0.870140699	0.00860655
ZNF680	2272	2198	1917	1209	-0.515324745	0.00925922
LOC283922	418	365	278	244	-0.583554143	0.01003835
C19orf52	469	476	570	846	0.584314508	0.00748897
TMEM70	644	604	743	1091	0.554366571	0.00756964
GPRC5C	259	278	335	525	0.67871484	0.00765379
TMOD2	470	436	309	317	-0.533358946	0.01077587
SPRED3	105	94	158	198	0.835329178	0.00807842
HIGD2A	1247	1233	1367	2283	0.557196485	0.00829996
SERPINE1	102	93	169	175	0.819874159	0.0084421
GPR157	30	22	56	80	1.379645511	0.00848026
PPP2R1A	7632	7151	7957	13097	0.5101071	0.00857116
FAM195B	514	422	561	864	0.605864255	0.00858745
MUL1	582	576	699	974	0.531154104	0.00858751
NECAB3	584	505	653	959	0.565540394	0.008664
SLC25A33	203	193	237	445	0.783780519	0.00871677
PMFBP1	98	158	69	60	-0.985187183	0.01112711
POLR2J2	138	144	96	32	-1.135084349	0.01138661
COMMD5	507	431	570	830	0.578312834	0.00882544
CALY	138	79	177	223	0.878199627	0.0091034
POU3F4	0	0	9	9	5.491251024	0.00912774
NAT14	652	660	735	1245	0.59262134	0.00929079
GSTT1	397	326	494	564	0.548394072	0.00932115
EME2	537	423	267	367	-0.598388316	0.01142631
ZNF431	607	630	489	375	-0.517751924	0.0115993
RPL13AP6	5	20	9	115	2.312346452	0.00934578
NEIL3	240	278	180	143	-0.681293243	0.01166625
TMEM175	242	181	294	382	0.674071905	0.00957765
NCAM1	11	4	0	0	-5.530638123	0.01192509
CITED4	112	93	170	189	0.803507485	0.0095892
ARPC3	961	1018	1109	1768	0.539343675	0.00964547
LOC202181	463	438	343	271	-0.554165885	0.01205855
AVPI1	330	311	412	545	0.576823551	0.0098863
ABCB4	69	56	25	25	-1.310064514	0.01213901
MFSD5	660	598	758	1033	0.509378616	0.01022346
LOC729513	159	172	84	106	-0.805393642	0.01227241
SCN5A	103	98	47	53	-1.007799546	0.01229222
ATP8B3	162	152	94	87	-0.79807311	0.01321932

TPST1	373	493	584	675	0.539905194	0.01030909
C17orf103	504	412	612	694	0.511512669	0.01031954
KRCC1	1040	1049	882	593	-0.501596016	0.01338552
COL11A2	328	324	223	214	-0.577116694	0.0136641
HBE1	2651	2542	2810	4555	0.503983944	0.01049277
PDF	89	71	112	202	0.964182815	0.01063334
C2orf84	18	9	1	1	-3.829287761	0.013791
CDH19	229	290	171	157	-0.659242156	0.01394578
CMYA5	105	69	40	40	-1.10545076	0.0140633
LOC729737	342	387	284	184	-0.635234174	0.01488401
RSPO3	128	101	68	50	-0.948183906	0.01528793
BAIAP2L2	42	56	130	79	1.105276029	0.01088223
LOC644649	117	145	91	31	-1.103975489	0.01564547
GPR172A	637	663	696	1308	0.622943135	0.0111763
SNORA72	0	0	8	9	5.415643279	0.01123666
FAM43B	136	150	213	253	0.700961575	0.01128431
FBXL15	253	225	313	424	0.622406258	0.01150788
NUBP2	717	790	868	1289	0.517791676	0.01151651
AHRR	19	20	51	54	1.452643614	0.01175229
ZNRD1	463	520	587	846	0.542927984	0.01182391
TRAPPC6A	126	149	192	269	0.743641246	0.01184917
OBFC2A	29	23	5	7	-2.104678067	0.01584042
SFT2D1	444	434	516	780	0.56159666	0.01258445
FAM43A	271	301	419	412	0.538993806	0.01280461
SPATA2L	817	819	898	1485	0.542169194	0.01299154
BACE1-AS	31	35	59	94	1.213717507	0.01306753
PHYHIP	74	60	117	135	0.907768665	0.01307443
GRRP1	231	153	269	351	0.692082253	0.01326871
TMEM183A	311	263	198	177	-0.610559354	0.01597493
PRAMEF2	64	64	109	133	0.916499332	0.01354136
ANKRD19P	247	249	188	113	-0.721152405	0.0162118
LOC389765	327	254	188	191	-0.617043695	0.0163109
PRSS23	263	206	337	371	0.5916151	0.01379151
LOC338799	604	433	356	361	-0.532211642	0.01712276
UQCRQ	1655	1492	1682	2821	0.516820017	0.01387233
GFI1	0	0	6	10	5.34118964	0.01390222
PLEKHF1	106	96	170	172	0.761254407	0.01415753
DECR2	326	310	362	628	0.636781637	0.01457169
C20orf27	872	842	933	1545	0.531164215	0.01463469
HMGN5	1519	1417	1304	670	-0.57217274	0.01736916
ZCRB1	2420	2843	2352	1321	-0.518334256	0.01829831
KDM4DL	117	159	184	284	0.761211025	0.0147022
DUSP15	64	36	94	115	1.04629247	0.01488154

FLJ43663	397	393	296	254	-0.523032474	0.01855356
LRRCC1	484	494	396	280	-0.533416163	0.01899274
DLEU2	151	158	103	77	-0.779534053	0.01899511
IL27RA	835	539	926	1044	0.518578496	0.0150477
RELB	277	302	384	456	0.536079546	0.01551454
UBE2D4	158	146	239	236	0.645366724	0.01566948
BCAS4	447	451	524	779	0.53596964	0.01570866
SRPX2	117	97	64	49	-0.920049653	0.02017157
LOC442459	22	26	49	70	1.315134705	0.01582128
FBXL14	527	457	561	883	0.553753737	0.01586127
NDN	1041	1236	1247	1983	0.504274727	0.01617058
ARL11	0	0	14	2	5.379398812	0.01632775
DET1	91	129	192	178	0.752135213	0.01649553
SYT4	48	66	20	27	-1.281795633	0.02069776
C1orf70	1	2	19	12	3.244402527	0.01675823
GATSL3	91	103	144	190	0.778176002	0.01686387
FAM180B	12	9	1	0	-4.519280143	0.02104342
SIRT6	297	265	367	451	0.541791543	0.0171924
MBD3L3	1	3	13	20	2.97063883	0.01723692
BANF1	1126	1109	1188	1993	0.50842644	0.0175614
OGFOD2	292	293	349	527	0.582170088	0.01768938
ALOX15B	0	0	8	7	5.240021016	0.01781448
CCDC150	529	518	436	283	-0.541696248	0.0215413
LMO7	255	282	211	121	-0.691412019	0.02154917
ITGB1BP2	63	42	27	13	-1.407546317	0.02178742
PLAC9	0	0	8	7	5.240021016	0.01781448
LOC10050566	132	132	88	60	-0.832099058	0.02208706
GSTZ1	420	377	480	661	0.51901592	0.01788702
PMS2CL	233	298	168	180	-0.605219261	0.022265
HES6	602	593	612	1208	0.606844741	0.01791759
PPAPDC3	199	177	212	415	0.735144195	0.01795399
MIR4720	0	0	4	12	5.26838876	0.01819723
ZMAT1	383	333	279	206	-0.560797354	0.02269193
ZNF837	100	105	147	201	0.758341087	0.01838761
MYEOV2	486	572	542	1094	0.62790785	0.01867083
SLC43A2	585	610	664	1044	0.515702652	0.01900715
PYCRL	299	295	356	523	0.56290453	0.01941752
FAM166A	20	5	0	2	-3.740319043	0.02280813
PRG4	631	796	618	322	-0.60074622	0.02300615
PCDH12	27	9	54	48	1.508198387	0.01941777
C8orf77	61	15	12	11	-1.783243228	0.02432911
PUSL1	241	224	284	423	0.604452645	0.01949702
FAM171A2	506	489	555	882	0.530488326	0.01996695

FCGBP	34	33	65	80	1.106056075	0.02078713
MIR3687	39	38	69	328	2.349167799	0.02091791
CORO7	87	79	109	193	0.85726904	0.02107344
HIST1H2BE	0	0	5	9	5.161681667	0.02184847
DGCR6L	178	144	227	270	0.626250949	0.02186161
MECR	314	323	397	509	0.508369744	0.02188252
GPR172B	0	0	6	8	5.155636223	0.02189497
VSIG1	7	5	0	0	-5.219691686	0.02445329
ITM2A	318	333	378	577	0.552132328	0.02231715
SH3TC1	76	100	123	185	0.810958088	0.02235454
TMEM158	332	313	383	553	0.536487178	0.02243347
SENP3-EIF4A	30	4	2	3	-2.886885599	0.02489391
GRHL3	107	77	153	159	0.761499475	0.02255913
NCRNA00235	0	0	4	11	5.173760911	0.02256051
TMEM121	106	156	159	291	0.782149143	0.02263247
SLC17A6	250	234	180	136	-0.615525589	0.02554941
DEXI	286	354	368	588	0.580365298	0.02289892
HIST1H2AE	69	90	139	135	0.792177867	0.02314942
GABRA2	315	327	398	513	0.50368768	0.02329325
EFNA3	410	577	383	302	-0.525584176	0.02644748
HDHD3	349	335	386	623	0.559727775	0.02341857
LOC148189	107	105	156	191	0.705391131	0.02349318
ABCB9	228	232	295	378	0.550923271	0.02355541
ELOVL3	69	57	101	128	0.864114346	0.02398491
BCDIN3D	186	219	247	375	0.619352083	0.02400132
NME6	183	169	231	304	0.602947167	0.024129
NDUFB6	564	682	664	1144	0.536280011	0.02427717
POLD4	399	299	372	711	0.632198406	0.02455463
C6orf154	66	71	115	127	0.814249919	0.02470576
LFNG	35	40	67	88	1.043383095	0.02482995
C8orf48	9	3	0	0	-5.207484762	0.02677568
ABCA10	389	345	279	235	-0.512014268	0.02680609
SHROOM2	264	294	426	365	0.503573737	0.02490737
PPAP2C	99	136	188	190	0.68761804	0.02502445
NMB	168	114	177	291	0.72740971	0.02512473
TMPRSS15	31	19	84	40	1.305170463	0.02525691
KIAA0040	58	27	17	13	-1.485225877	0.02736346
BMS1P4	154	146	88	94	-0.715412468	0.02749317
MUC1	272	270	208	153	-0.585579239	0.02754795
FBXW9	265	244	280	504	0.623010748	0.02609494
EIF5AL1	238	193	258	401	0.611222733	0.02613253
ZDHHC24	163	171	202	323	0.648451181	0.02672348
ZMYM6NB	325	355	399	574	0.515541081	0.02673774

SNX11	373	337	391	653	0.555221947	0.02720498
NPPA-AS1	38	66	22	21	-1.283834485	0.02807939
LOC155060	257	277	169	193	-0.559525618	0.02842903
SYT14	52	38	15	20	-1.349158534	0.02890388
TMEM208	340	334	381	599	0.539161508	0.02720814
TBR1	0	0	13	1	5.20472086	0.02730244
LYSMD4	224	202	277	347	0.548668183	0.0276066
C15orf52	17	13	0	4	-2.97279122	0.02910646
EDAR	0	0	4	9	5.066025216	0.0276735
GLI4	314	237	343	463	0.546809232	0.02768757
PKI55	169	152	105	94	-0.690598814	0.02936624
SS18L2	485	509	522	923	0.53926552	0.02788377
CEBPA	233	144	238	357	0.658447508	0.02920453
IFI27L1	155	152	217	244	0.588423977	0.02937955
LOC645166	10	7	24	34	1.769274544	0.02941719
NAALADL2	45	83	26	31	-1.169449892	0.02950523
C19orf79	179	125	301	187	0.68465857	0.0297342
SEPT5-GP1BE	102	115	67	55	-0.835787376	0.02953864
TRIM64B	76	40	113	104	0.912309597	0.03014088
ANAPC11	665	582	621	1206	0.550399142	0.03095335
C1QTNF3	101	120	66	59	-0.823505724	0.03026797
LRRC69	59	38	15	24	-1.313408923	0.03063521
ANKRD44	164	152	94	103	-0.682976777	0.03140787
UCN	7	8	20	34	1.83838263	0.03136135
PSMB10	149	185	190	340	0.670195202	0.03219391
LOC10013009	245	200	272	386	0.561423335	0.03253252
AIDA	269	272	267	585	0.65396747	0.03264714
NAT1	133	126	192	204	0.610776544	0.03282134
UCKL1-AS1	10	7	31	26	1.735108484	0.03294877
NR2C2AP	233	155	226	386	0.656098907	0.03319923
MIR17HG	316	471	239	298	-0.550608246	0.03165407
KAT2B	194	204	285	286	0.520360304	0.03344175
LOC283116	83	103	167	137	0.708807047	0.03353389
DDX11L2	18	23	4	6	-2.12118366	0.03210612
SRGAP3	142	167	224	237	0.577051062	0.03355663
TRANK1	33	44	81	71	0.982741215	0.03367468
SNORD50B	75	53	38	19	-1.157166184	0.03309541
CCDC58	929	958	864	426	-0.547623541	0.0331477
NSUN5	338	259	319	585	0.597348976	0.03412523
LOC10028918	3	2	9	23	2.591180987	0.03429781
LOC729156	0	1	8	12	4.194756546	0.03449453
C9orf89	306	281	346	490	0.506465256	0.03450021
C17orf90	272	265	320	445	0.50970004	0.03493126

LOC10050706	317	298	243	178	-0.543616361	0.03321053
CCDC15	322	317	253	191	-0.526210982	0.03407574
LOC644838	0	0	4	8	4.956621318	0.03499956
TSPAN33	122	129	178	205	0.616880876	0.03515535
ANXA2P1	0	1	11	9	4.181887309	0.03524997
EMID1	156	147	179	294	0.641618089	0.03543315
CYB561D2	118	81	108	250	0.838457044	0.0354465
SNORD75	0	0	3	10	4.970552808	0.03630251
SNORA77	0	0	7	5	4.935616875	0.03644937
COL9A2	232	203	145	146	-0.582615685	0.03465326
PGBD5	80	70	123	127	0.736651298	0.03734677
GPR77	0	0	8	4	4.928527624	0.03782101
SLC2A8	222	205	247	386	0.564704452	0.0379759
DLL3	240	241	259	461	0.582495082	0.03866331
NUDT18	42	51	85	85	0.888352751	0.03889294
C6orf226	32	45	51	108	1.043789315	0.03956888
IL5RA	11	7	1	0	-4.282046761	0.03549658
FZD9	61	59	103	106	0.790441263	0.03987151
ANKRD5	57	70	109	110	0.780740019	0.03991987
C20orf29	124	159	162	289	0.673169818	0.04027958
TMCC3	88	99	149	148	0.666189491	0.04063625
LOC374443	89	116	142	186	0.674048942	0.0407897
CLDN11	46	70	99	107	0.830105716	0.04093322
NPAS1	159	129	194	238	0.588065394	0.04096511
PDRG1	527	425	496	862	0.511630964	0.04119126
ETV2	4	13	26	31	1.7221322	0.04122761
HIST1H3H	47	55	75	112	0.875078477	0.04131792
MIR941-1	7	16	1	2	-3.077029071	0.03676071
RDH14	119	91	161	169	0.645619187	0.04254991
PIGV	160	199	217	317	0.573673425	0.04310593
GPR146	51	47	14	27	-1.249250836	0.03700128
SGCA	30	42	57	87	0.997629428	0.0432946
TMEM99	92	88	131	156	0.67251549	0.04357089
LRRC28	230	191	251	362	0.540833519	0.04358575
AHR	5	7	29	15	1.911264978	0.04396162
RASSF7	196	165	215	322	0.57133001	0.04399337
FAM198B	166	197	246	276	0.523381091	0.04413464
ANGPT2	262	266	212	139	-0.585718888	0.0371625
MICAL2	103	90	151	151	0.648615799	0.04443471
TMSB15B	100	120	124	237	0.718750712	0.04447338
EXOC3L2	0	0	4	7	4.838239713	0.04460969
DHRS12	132	163	205	230	0.565177169	0.04462728
LOC144486	52	56	24	25	-1.128954926	0.03752724

LGALS3BP	95	80	130	149	0.678859935	0.04489736
FAM108A1	191	192	218	352	0.570439566	0.04497225
CCDC112	895	782	760	417	-0.509863756	0.03760629
MYBPH	14	0	0	0	-5.303434022	0.03827681
SQRDL	19	25	40	60	1.176928116	0.04582164
IMPG2	83	85	40	50	-0.888812999	0.03975504
VAV1	39	37	13	17	-1.361152785	0.03976663
LOC10050647	179	254	155	127	-0.614959353	0.03977796
FAM123A	0	0	13	0	5.105030291	0.04674614
DUXA	10	12	30	33	1.475900192	0.04706757
GAL3ST4	260	246	184	167	-0.529337693	0.03996161
FAM46B	98	87	119	179	0.691249925	0.04750861
VAT1L	24	19	6	5	-1.935014305	0.04016088
RFPL2	44	59	108	77	0.84887373	0.04791632
NBPF14	237	279	208	134	-0.591703135	0.0404246
MMP19	0	0	11	1	4.906665076	0.04827008
CRB1	7	10	0	1	-4.192858942	0.04092346
CDHR3	73	59	34	32	-1.00031931	0.04097502
TFAP2B	111	122	76	62	-0.752824282	0.04125419
EDNRB	0	13	0	0	-5.262567829	0.04149795
LOC10013119	91	141	65	69	-0.788928614	0.04162781
LAT2	24	11	4	4	-2.228368538	0.04204704
COL6A4P1	10	1	0	0	-5.069964207	0.04215963
TRIM34	10	1	0	0	-5.069964207	0.04215963
EYA1	53	79	39	25	-1.047156303	0.04230709
DNAI1	20	11	4	1	-2.480215737	0.04350181
KCTD19	32	27	9	12	-1.5387413	0.04354221
CCDC154	22	63	18	13	-1.418665565	0.04393014
FKBP11	430	460	241	388	-0.502829189	0.04447165
EMCN	10	13	0	3	-3.049525566	0.04493036
SNORA40	127	139	101	56	-0.762107723	0.04496352
LOC10027222	144	112	84	73	-0.708303704	0.04665002
KLRAP1	71	135	58	56	-0.856376249	0.04737671
ITGA4	90	96	62	42	-0.829136584	0.04791302
KCNJ4	165	123	193	237	0.577887166	0.04891929
ZNF836	201	216	168	103	-0.617176018	0.04792594
CCDC78	112	80	54	56	-0.808746882	0.04880771
IFI35	157	145	228	210	0.536501085	0.04965225
C9orf43	11	5	32	21	1.660327762	0.04975117

Supplementary Table S2

C20orf27
OGFOD2
UPF3A
LAGE3
PEX16
KRCC1
AHRR
GPR157
SH3D21
ANKRD36
GRRP1
NINJ1
NBPF14
FKBP11
YBX2
ARL11
ELOVL3
PPAP2C
EMID1
GRHL3
FAM173A
ISG15
GPR172A
PEMT
COL9A2
C15orf52
TMED9
AGTRAP
ZNF107
EME2
C2orf84
LFNG
PROCA1
MMP11
COL11A2
FAM176B
SLC43A2
GSDMB
TST
CHCHD10
COL27A1
KAT2B

YIF1B
ZNF837
CHID1
NPAS1
ISOC2
RHBDD2
NCRNA00202
C20orf29
TMEM158
SGCA
HES6
HK2
GPR146
NECAB3
SRGAP3
HSPA1B
NRBP2
FDXR
DUSP15
TLCD1
PSMB10
MAD2L2
SCN5A
PHYHIP
SLC26A10
C11orf2
C1QTNF3
NCAM1
PI4KAP1
COL7A1
CMTM3
AHNAK
SLC35B1
TP53I3
UCP2
LMO7
ABCB9
MAMDC4
NDUFS3
MAP2K3
MFSD3
C17orf70
FAM43B

CLDN11
KIAA1797
SNORD57
C6orf154
KDM4DL
DLL3
CCDC154
ALOX15B
PRSS23
GPR172B
C9orf89
TCEB2
GLS2
DPCD
DNAI1
CILP2
UBE2D4
MMP19
RASSF7
HPS6
KCND1
PUSL1
ZNF836
CLP1
ZDHHC24
ZNF92
TXNRD2
ALPPL2
LAT2
CDHR3
SH3TC1
FAM195B
S100A4
C7orf59
MICAL2
CD81
C1orf70
INPP5D
SNAP47
F11R
NME3
GLI4

Supplementary Table S3

Gene	Primer sequence (5'-3')
CDKN1A-F	CGATGGAACTTCGACTTTGTCA
CDKN1A-R	GCACAAGGGTACAAGACAGTG
FDXR-F	CTGAGGCAGAGTCGAGTGAAG
FDXR-R	CCCGAAGCTCCTTAATGGTGA
RPS27L-F	TTACTACATCCGTCCTTGGAAGA
RPS27L-R	GCATGGCTGAAAACCGTGG
TP53I3-F	GGAGGACCGGAAAACCTCTAC
TP53I3-R	CCTCAAGTCCCAAAATGTTGCT
ANXA1-F	CTAAGCGAAACAATGCACAGC
ANXA1-R	CCTCCTCAAGGTGACCTGTAA
PLTP-F	AAGAGCGGATGGTGTATGTGG
PLTP-R	ATGGGGAGTCAATCACTGCTG
SESN2-F	TCTTACCTGGTAGGCTCCCAC
SESN2-R	AGCAACTTGTTGATCTCGCTG
NINJ1-F	TCAAGTACGACCTTAACAACCCG
NINJ1-R	TGAAGATGTTGACTACCACGATG
DOK3-F	CGGCTCCGACAAGATACTTCT
DOK3-R	CGTCATCGGTTCTCTCTCCTT
PDK1-F	CTGTGATACGGATCAGAAACCG
PDK1-R	TCCACCAAACAATAAAGAGTGCT
ANKRD36-F	CCGCCTTTATTCACGCATACG
ANKRD36-R	AGGAGGTCGCTCAGAAGATTC
SLC26A10-F	GGCCGAACTGACCATCTCC
SLC26A10-R	CCGGTCTCGGAATCTCACG
CFH-F	CACACAAGATGGATGGTCGC
CFH-R	GGATGGCAGGCAACGTCTAT
DNHD1-F	GTGAATCCCGAGCACTACATC
DNHD1-R	TCATCGTCTCGCTACCTTCCA
MYBL1-F	AGGCAAGCAGTGTAGAGAAAGA
MYBL1-R	CGATTTCCCAACCGCTTATGT
PRRT2-F	TTCTGTCTGAGAGTGTAGGGG
PRRT2-R	CAGGCTACCTCGGGGAGAT
HK2-F	TGCCACCAGACTAAACTAGACG
HK2-R	CCCGTGCCCACAATGAGAC
STC2-F	ACAGGTTCGGCTGCATAAGC
STC2-R	GAGGTCCACGTAGGGTTCG
CCDC18-F	AGGAAGACCGTTGCATTGG
CCDC18-R	TGCAGTCAGGTTTGCATGAAG
AGER-F	GTGTCCTTCCCAACGGCTC
AGER-R	ATTGCCTGGCACCGGAAAA
SOX2-F	TGCGAGCGCTGCACAT
SOX2-R	TCATGAGCGTCTTGGTTTTCC
CD133-F	GAACAAGTTTACAGTGACTGC

CD133-R	TGCGTTGAAGTATCTTTGACG
OCT4-F	GGAGGAAGCTGACAACAATGAAA
OCT4-R	GGCCTGCACGAGGGTTT
NANOG-F	ACAACTGGCCGAAGAATAGCA
NANOG-R	GGTTCCCAGTCGGGTTCAC
GAPDH-F	TCGACAGTCAGCCGCATCT
GAPDH-R	CTAGCCTCCCGGGTTTCTCT
